# Probing Structural Evolution and Charge Storage Mechanism of NiO_2_H*_x_* Electrode Materials using In Operando Resonance Raman Spectroscopy

**DOI:** 10.1002/advs.201500433

**Published:** 2016-02-23

**Authors:** Dongchang Chen, Xunhui Xiong, Bote Zhao, Mahmoud A. Mahmoud, Mostafa A. El‐Sayed, Meilin Liu

**Affiliations:** ^1^ School of Materials Science and EngineeringCenter for Innovative Fuel Cell and Battery TechnologiesGeorgia Institute of Technology771 Ferst DriveAtlantaGA30332‐0245USA; ^2^ Laser Dynamics LaboratorySchool of Chemistry and BiochemistryGeorgia Institute of Technology901 Atlantic DriveAtlantaGA30332‐0400USA

**Keywords:** battery, nickel hydroxide/oxo‐hydroxide, reaction mechanisms, resonance Raman spectroscopy, supercapacitor

## Abstract

**In operando resonance Raman spectroscopy** suggests quantitative correlation between phonon band properties and the amount of charge storage of high‐energy density NiO_2_H*_x_* battery/pseudocapacitive material. Comparing the spectroscopic evolution using different electrolytes reveals the contributions of breaking/formation of O–H bonds and insertion/extraction of cations to electrochemical charge storage of NiO_2_H*_x_*.

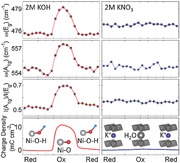

The demand for clean, affordable, and reliable energy storage devices has greatly inspired extensive global research on advanced battery/capacitor technologies.^1^, ^2^ In particular, nickel hydroxides/oxo‐hydroxides compounds (NiO_2_H*_x_*) are among the most attractive materials for energy storage because of their unprecedented theoretical capacity (e.g., ≈3000 F g^−1^ for capacitors and ≈450 mAh g^−1^ for batteries) and superior stability within a wide range of potential due to low equivalent mass and a broad oxidation state window of Ni (Ni(II)–Ni(IV)).^3^, ^4^, ^5^, ^6^ In recent years, various nanostructured electrode materials (e.g., low dimension nanomaterials and heteroatom‐doped 3D nanostructures),^7^, ^8^, ^9^, ^10^ have been created to enhance the electrochemical performance,^11^, ^12^, ^13^, ^14^ resulting in larger capacity, higher rate capabilities, and longer cycling life. Despite the impressive progress on electrochemical performance achieved so far, a fundamental understanding of the structural evolution, charge storage mechanism, and the contribution of redox reaction to capacity in NiO_2_H*_x_* is still lacking, due primarily to the complex crystalline structures of the redox active NiO_2_H*_x_* under cycling conditions.^15^ The basic structural element of the NiO_2_H*_x_* crystal is the NiO_2_ layers composed of edge‐sharing NiO_6_ octahedra. On one hand, hydrogen atoms can be bonded to the oxygen atom forming O—H bonds in the layered framework.^15^ On the other hand, the interlayer spacing can accommodate different types of species (e.g., ions and water molecules as schematically shown **Figure** **1**a).^15^ The stoichiometry of H and interlayer ions are in accordance with the oxidation state of Ni, thus leading to a variety of polymorphs: when *x* is close to 2 and interlayer species are incorporated, the structure is named as α‐Ni(OH)_2_;^16^, ^17^ the extraction of interlayer species from α‐Ni(OH)_2_ leads to a closely packed layered structure, denoted as β‐Ni(OH)_2_;^17^, ^18^, ^19^ the oxidized form of α‐Ni(OH)_2_ is named as γ‐NiOOH (with interlayer species);^5^, ^18^, ^19^, ^20^ the extraction of interlayer species from γ‐NiOOH lead to the structure called β‐NiOOH.^15^, ^20^ The completely dehydrogenated form of NiO_2_H*_x_* with large amount of cation incorporation (e.g., Li_1−_ *_δ_*NiO_2_) can also be categorized in this class of material.^15^, ^21^, ^22^ These types of polymorphs can be readily interconverted from one to another.^18^, ^19^

**Figure 1:**
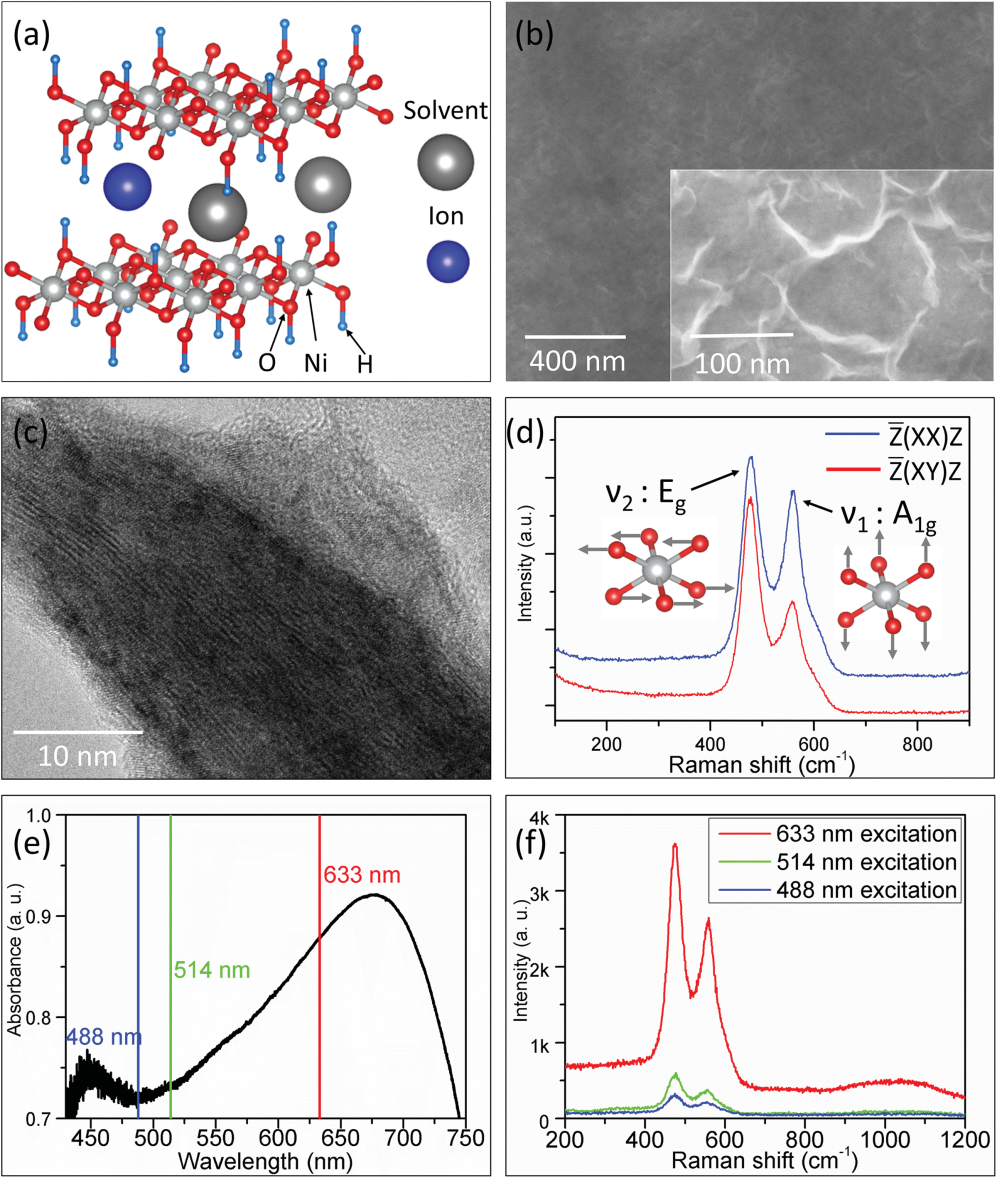
a) Schematic sketch of NiO_2_H*_x_* layered structure showing bonded hydrogen and interlayer species (ions and solvent molecules). b) Scanning electron microscope (SEM) images of NiO_2_H*_x_* thin film model electrode. c) TEM image of the scraped NiO_2_H*_x_* thin film, d) Raman spectra of NiO_2_H*_x_* thin film model electrode under and polarization configurations. The corresponding band assignments (E_g_ and A_1g_) and the sketches of the two bands are also shown. e) UV–vis absorption spectrum of NiO_2_H*_x_* thin film model electrode marked with available excitation laser wavelengths. f) Raman spectra of NiO_2_H*_x_* thin film model electrode using different excitation lasers with equivalent laser power of 4 mW.

Despite the complicated polymorphs of NiO_2_H*_x_*, the basic structure (Figure 1a) on which various polymorphs are based has two possible mechanisms to store charge (i.e., to change the oxidation states of Ni); the breaking/formation of O—H bond and insertion/extraction of electrolyte cations. On one hand, since the energy storage of NiO_2_H*_x_* is primarily operated in KOH electrolyte, the OH^−^‐assisted breaking/formation of O—H bond is expected to play a significant role.^5^, ^11^, ^23^ On the other hand, a lot of researches suggested cation insertion/extraction can also contribute considerably to charge storage,^24^, ^25^, ^26^ since it has been revealed that the stoichiometry of NiO_2_H*_x_* can accommodate large amount of cations between the layers (e.g., Na_0.32_ H_0.22_(H_2_O)_0.25_NiO_2_ and other similar forms).^5^, ^18^, ^27^, ^28^ Cation insertion/extraction is also responsible for charge storage of other types of layer‐structured pseudocapacitive and battery materials (e.g., layered MnO_2_‐based pseudocapacitor and LiCoO_2_/LiNiO_2_‐based Li‐ion battery cathodes).^22^, ^29^, ^30^ Therefore, the detailed contribution of both breaking/formation of O—H bond and cation insertion/extraction should be systematically evaluated. More importantly, it is imperative to quantitatively correlate the charge storage with the structural features of NiO_2_H*_x_* in order to gain a fundamental understanding of the charge storage mechanism.

Raman spectroscopy, which is based on inelastic scattering of phonon modes and photons, is ideally suited for revealing the vital structural information of NiO_2_H*_x_* through the properties of phonon bands.^31^ Moreover, by carefully tuning the Raman exication laser frequency close to the energy of electronic state transition, resonance Raman effect can provide unparalleled sensitivity to probing subtle structural evolution of materials. Such resonance enhancement effect has been proven vital to unambiguously probing fundamental properties of various materials (e.g., graphene and graphene‐based derivatives).^32^, ^33^, ^34^ In fact, normal Raman spectroscopy has been used to study the properties of NiO_2_H*_x_* such as charge storage, catalysis, and other types of structural transformations.^35^, ^36^, ^37^, ^38^, ^39^ However, a clear quantitative correlation between the structural features and the electrochemical properties is still lacking. In this work, relying on resonance enhancement, we used in operando resonance Raman spectroscopy to evaluate the charge storage contribution of two possible redox mechanisms for NiO_2_H*_x_* by adjusting properties of electrolyte solutions. Key phonon properties were quantitatively analyzed and correlated with the amount of stored charge, providing important insight into the mechanism of charge storage and scientific basis for knowledge‐based design of better electrode materials.

In this study, a thin film model electrode was fabricated by electrochemically oxidizing the bare Ni foil (Supporting Information). During the extensive oxidation of Ni, nickel hydroxides would be gradually formed and eventually become γ‐NiOOH which is the polymorph with considerable amount of incorporated cations and water molecules and an oxidation state around Ni(III) (Supporting Information).^19^, ^38^, ^40^ Figure 1b shows the morphology of the model electrode, indicating a flat surface which is beneficial for acquisition of Raman spectra and unambiguous correlation of structural properties and electrochemical behaviors. A closer view of the surface (Figure 1b, inset) clearly shows the flake‐like morphology, similar to the typical morphologies of NiO_2_H*_x_* reported elsewhere.^11^, ^13^ The transmission electron microscope (TEM) image of the scraped thin film clearly displays the layered‐like fringes, proving the formation of layered NiO_2_H*_x_* framework (the selected area electron diffraction (SAED) pattern is shown in the Supporting Information).

Figure 1d shows the Raman spectrum of the thin film model electrode. Two Raman bands can be clearly observed and match very well with Raman spectrum of γ‐NiOOH.^36^, ^38^, ^41^ The two bands (labeled as ν_1_ band and ν_2_) band can be assigned as the polarized A_1g_ mode and depolarized E_g_ mode of the NiO_2_ framework respectively based on group theory (Supporting Information). Polarized Raman analyses revealed that the relative intensity of A_1g_ band decreased significantly after the polarization configuration was changed from to , validating the band assignments (Supporting Information) and formation of NiO_2_ framework (to be specific, γ‐NiOOH). More importantly, since these two phonon modes are directly related to the lattice vibration of NiO_2_ framework, the structural changes due to charge storage (e.g., Ni—O bond length, electron cloud distribution, and structural disorder) will inevitably lead to the evolution of phonon properties, including phonon hardening/softening, polarizability change, and degeneration of symmetry, which will be observed as evolutions of band positions, band intensities, and band profiles experimentally. The quantification of these phonon properties provides solid basis to correlate the structural changes with charge storage contribution unambiguously, which makes in operando Raman spectroscopy perfectly suited for this study.

To optimize the sensitivity of probing the subtle changes in phonon bands during in operando experiments, we examined the light absorption properties and resonance Raman effect of NiO_2_H*_x_* model electrodes. Figure 1e shows the absorption spectrum of a NiO_2_H*_x_* model electrode in the visible light range. An absorbance maximum was clearly observed in the range of 650–700 nm. Among the wavelengths of commonly used lasers in the visible range, the wavelength of He–Ne laser (633 nm) is the closest to the absorbance maximum (Figure 1e), which is closer than those of the Argon ion lasers (488 and 514 nm). Consequently, much stronger resonance enhancement was observed under excitation of 633 nm laser than 488 or 514 nm laser of equivalent laser power (Figure 1f), despite the fact that normal Raman scattering efficiency is proportional to the fourth power of the excitation laser frequency (I ∝ *λ* ^−4^). Because of the strong resonance enhancement under 633 nm laser excitation, the sensitivity and specificity of in operando Raman spectroscopy has provided us unambiguous correlation between phonon and electrochemical properties.

The in operando Raman spectroscopic evolution was tested first in a 2 m KOH aqueous electrolyte, which is aimed to evaluate the structural changes of NiO_2_H*_x_* in general charge storage applications. It is noted that in KOH electrolyte both cation insertion/extraction (K^+^ ions) and breaking/formation of O—H bond are likely to contribute to the charge storage process. **Figure** **2** shows the in operando resonance Raman spectroscopic evolution along with the CV profile in the potential window of 0–0.5 V. It is observed that a pair of redox peaks appeared in the range of 0.3–0.5 V (vs Ag/AgCl), which is in accordance with the electrochemical behaviors of most reported works of NiO_2_H*_x_*.^11^, ^42^ This CV profile can be well maintained as the scan rate increased up to 500 mV s^−1^ (Supporting Information). More than 80% of the charge storage capacity (261 mAh g^−1^, Supporting Information) was retained when the current density was increased from 0.025 to 5 mA cm^−2^ (2.1 to 427 A g^−1^ with respect to mass of active material, Supporting Information).

**Figure 2:**
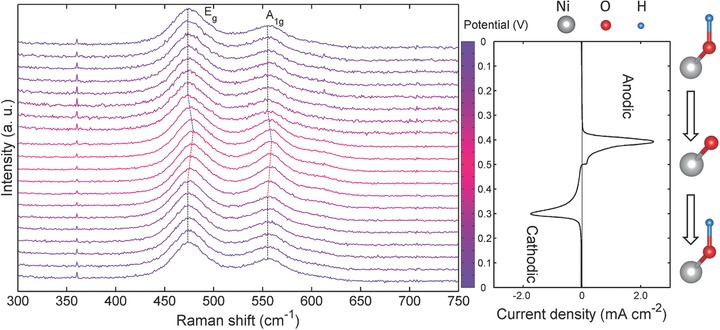
In operando Raman spectroscopic evolution of NiO_2_H*_x_* thin film model electrode operated in a 2 m KOH aqueous electrolyte. The corresponding CV profile (10 mV s^−1^) is shown on the right panel. The potential of each spectrum is indicated by the color bar located between the spectra and the CV curve. The color bar is separated to 20 grids. Each grid represents a potential interval of 0.05 V, over which, the spectrum was acquired during the in operando measurements. The red shifts and blue shifts of A_1g_ mode and E_g_ mode are marked on the spectra. A schematic sketch of breaking/formation of O‐H bond is also shown to illustrate the redox behavior on the basis of quantitative analyses.

The in operando Raman spectra were acquired with an interval of 0.05 V during CV test. First, no new bands were observed, proving no extra phases were produced during charge storage. Second, it is clearly shown that the Raman spectra didn't present significant evolution in the potential range where no obvious charge storage is observed (0–0.35 V in anodic process, 0.3–0 V in cathodic process). Third, however, in the potential range between 0.35–0.5 V in anodic process, the energy of E_g_ mode, denoted as *ω*(E_g_), was blue shifted from 474 to 480 cm^−1^. Similarly, *ω*(A_1g_) was blue shifted to 554 to 558 cm^−1^. The intensity ratio, denoted as I(A_1g_)/I(E_g_), increased from 0.50 to 0.69. These phenomena clearly indicate massive positive charge storage lead to the stiffening of both A_1g_ mode and E_g_ mode, as a straightforward indication of shortening of Ni—O bond, and an increase of the polarizability of A_1g_ mode which is due to the change of electron cloud distribution within NiO_2_ layer. Reversibly, significant red shift of both A_1g_ mode and E_g_ mode and a decrease of I(A_1g_)/I(E_g_) were observed in the range between 0.5–0.3 V in cathodic process along with significant reductive current, which suggest the release of the stored positive charge restored the Ni—O bond length and electron cloud distribution within NiO_2_ layer. Under galvanostatic charge/discharge conditions, the evolution of Raman spectra was more gradual, not abrupt as seen under CV conditions, implying that the electrochemical current leads to structural changes (Figure S13, Supporting Information). Also, the Raman spectroscopic evolution remained the same as the electrolyte was changed from KOH to NaOH and LiOH, implying that the charge storage mechanism is independent of the types of the electrolyte cations studied (Figure S15, Supporting Information). In addition, we performed the reflectance measurement of the model electrode under in operando conditions (Figure S7, Supporting Information). The significant red shift of the reflectance profile (corresponding to blue shift of the absorption profile) qualitatively indicates the change of the oxidation state of Ni between the most oxidized/reduced states, consistent with the in operando Raman spectroscopic evolution mentioned above.

However, in KOH electrolyte, it cannot unambiguously distinguish the contribution of charge storage and structural evolution of possible redox mechanisms, due to the fact that the both cation insertion/extraction and breaking/formation of O—H bond are likely to contribute to the charge storage as mentioned above. Thus, to unambiguously unravel this issue, the in operando Raman spectroscopic evolution was performed in a neutral electrolyte (2 m KNO_3_) to evaluate the charge storage contribution from cation insertion, since the anion of the neutral electrolyte (NO_3_^−^) cannot be involved in the reaction of breaking/formation of O—H bond. **Figure** **3** shows the in operando Raman spectroscopic evolution along with the CV profile. The potential window used in 2 m KNO_3_ was shifted to higher potential range (0.5–1.0 V) compared to the potential window used in 2 m KOH electrolyte (0–0.5 V), since the stability window of NiO_2_H*_x_* will shift to higher potentials as PH decrease according to the Pourbix diagram.^38^, ^43^ It is clearly noted that the CV exhibited a rectangular‐like profile which can be maintained up to 500 mV s^−1^ (Supporting Information), similar with the case of layer‐structured pseudocapacitive MnO_2_,^29^ indicating cation insertion/extraction can contribute to charge storage of NiO_2_H*_x_*. For the Raman spectroscopic investigation, it is observed that the Raman bands were broadened as soon as the NiO_2_H*_x_* was immersed in 2 m KNO_3_ electrolyte (Figure S11, Supporting Information). This phenomenon also indicates the cation incorporation can take part in the charge storage of NiO_2_H*_x_*, since the interlayer cation can lead to structural disorder and band broadening effect consequently, which was observed for many other layer‐structured transition metal oxides.^29^, ^44^

**Figure 3:**
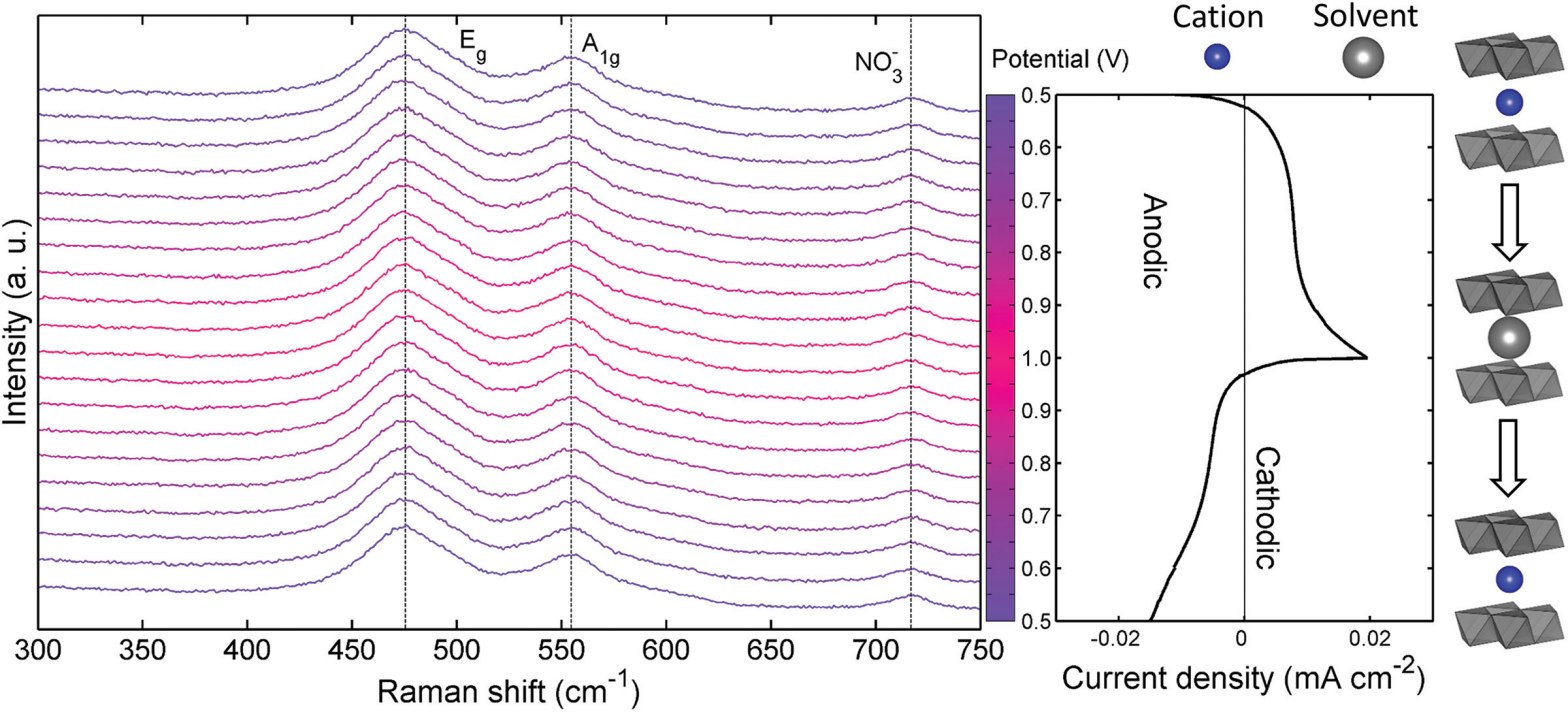
In operando Raman spectroscopic evolution of NiO_2_H*_x_* thin film model electrode operated in a 2 m KNO_3_ aqueous electrolyte. The corresponding CV profile (10 mV s^−1^) is shown on the right panel. The potential of each spectrum is indicated by the color bar located between the spectra and the CV curve. The color bar is separated to 20 grids. Each grid represents a potential interval of 0.05 V, over which, the spectrum was acquired during the in operando measurements. A schematic sketch of interlayer cation insertion/extraction is also shown to illustrate the redox behavior in KNO_3_ electrolyte on the basis of quantitative analyses.

However, it is obvious that the CV current density (Figure 3) was much lower than that of the CV tested in 2 m KOH electrolyte (Figure 2). Moreover, during the operation of the NiO_2_H*_x_*, the Raman spectra of NiO_2_H*_x_* didn't exhibit noticeable feature changes (Figure 3); *ω*(E_g_) and *ω*(A_1g_) were retained to be 476 and 554 cm^−1^, respectively, while I(A_1g_)/I(E_g_) was calculated to be 0.57. These experimental facts suggest the charge storage caused by cation incorporation can't cause significant evolution of phonon energies and phonon polarizabilities, implying the properties of Ni—O bond and electron cloud distribution within NiO_2_ layer remain static during cycling. Meanwhile, the static spectroscopic evolution is in accordance with the limited current density observed in the CV profile, which qualitatively indicates the cation incorporation cannot lead to massive charge storage proven by both electrochemical and structural analyses.

To quantitatively analyze the charge storage contribution of the two redox mechanisms, we performed quantitative Raman spectroscopic analyses to correlate the structural features and charge storage. The two Raman bands (A_1g_ and E_g_) were fitted using a Lorentzian profile (Supporting Information) and the stored charge density were calculated by integrating the CV profiles acquired during in operando experiments shown in Figures 2 and 3 (the integration is shown in Supporting Information). **Figure** **4** shows the energy of E_g_ mode, energy of A_1g_ mode, I(A_1g_)/I(E_g_), and the charge storage density as functions of the potential of WE for both KOH and KNO_3_ electrolyte. In the KOH electrolyte (Figure 4a–d), the evolution of *ω*(E_g_), *ω*(A_1g_), and I(A_1g_)/I(E_g_) are in excellent quantitative agreement with the profile of stored charge as a function of the potential, proving that the amount of charge storage is quantitatively associated to phonon energies and phonon polarizabilities. To be specific, in the potential range where no significant charge is stored/released, the phonon band properties remain largely static; as significant amount of charge is stored/released, the phonon band energy (*ω*(E_g_) and *ω*(A_1g_)) exhibit systematic stiffening/softening and band intensity ratio I(A_1g_)/I(E_g_) show systematic increase/decrease as a same profile of charge storage (Figure 4a–d). On the other hand, in KNO_3_ electrolyte, all of the band features basically retained the same values as functions of potential, also quantitatively consistent with the limited stored charge (about 4% of the stored charge in KOH electrolyte based on the enlarged plot). Thus, it can be concluded that the structural features and charge storage in KOH electrolyte shown in Figure 4 are essentially contributed by the breaking/formation of O—H bond (as marked in Figure 2) with minor contribution from cation insertion/extraction (marked in Figure 3).

**Figure 4:**
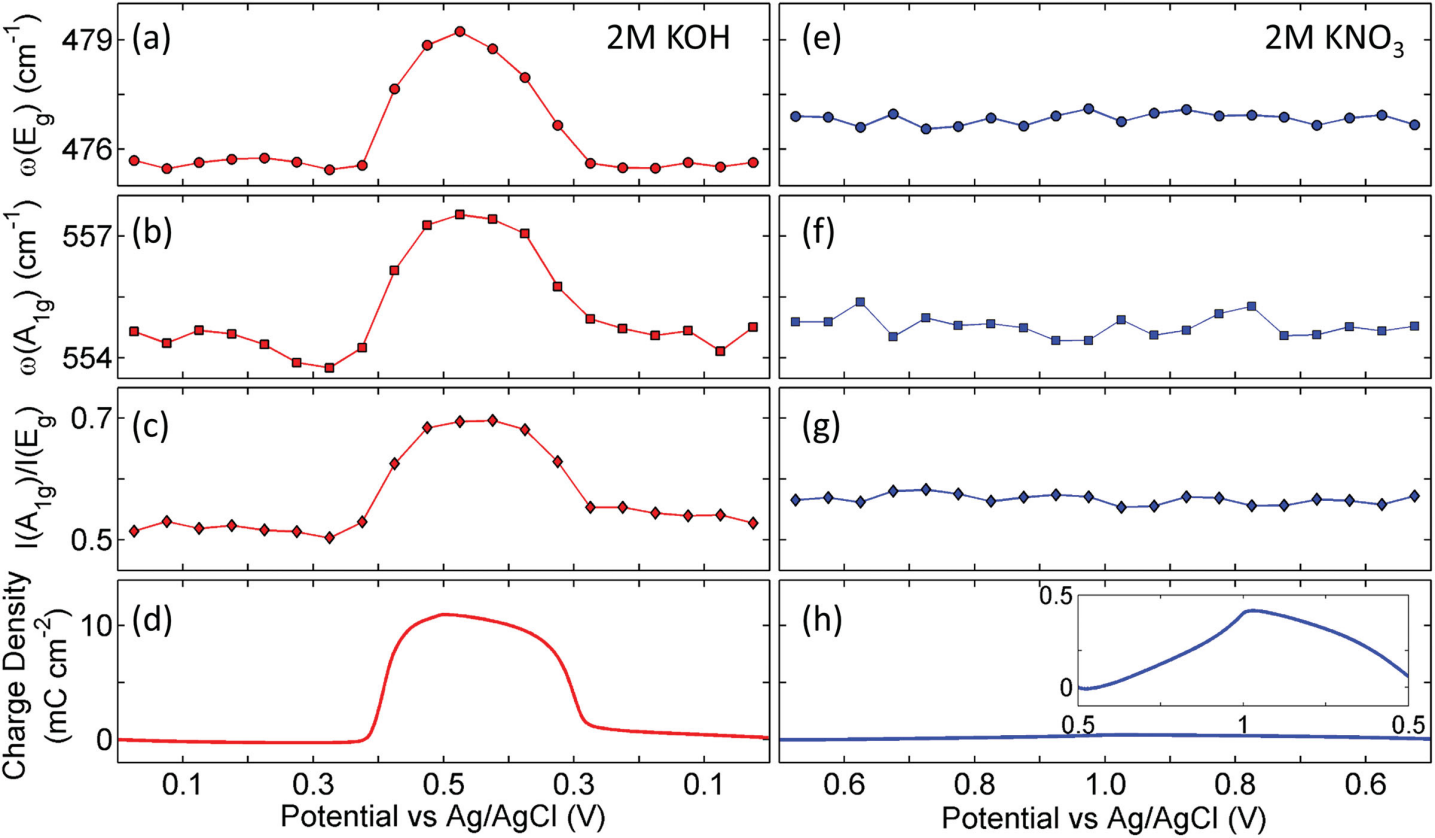
Quantitative correlation between key spectroscopic features and stored charge density of NiO_2_H*_x_* model electrode when 2 m KOH and 2 m KNO_3_ were used as electrolyte. a–d) The energy of E_g_ mode (i.e., *ω*(E_g_)), energy of A_1g_ mode (i.e., *ω*(A_1g_)), intensity ratio of A_1g_ and E_g_ (i.e., I(A_1g_)/I(E_g_)), and stored charge density as functions of WE potential when KOH was used as electrolyte, respectively. e–h) The values of *ω*(E_g_), *ω*(A_1g_), I(A_1g_)/I(E_g_), and stored charge density as functions of WE potential when KNO_3_ was used as electrolyte, respectively.

In conclusion, we have performed a systematic in operando resonance Raman spectroscopic study of thin‐film NiO_2_H*_x_* model electrodes to investigate the contributions of two possible redox mechanisms of NiO_2_H*_x_* during cycling. Depending on resonance enhancement effect, it is found that the phonon properties of NiO_2_H*_x_*, including *ω*(E_g_), *ω*(A_1g_), and I(A_1g_)/I(E_g_), exhibited systematic evolution along with massive redox charge storage in 2 m KOH electrolyte, whereas limited charge storage and spectroscopic evolution were observed in 2 m KNO_3_ electrolyte. Quantitative Raman band analyses indicate that phonon properties (*ω*(E_g_), *ω*(A_1g_), and I(A_1g_)/I(E_g_)) show strong quantitative dependence on charge storage, confirming that the breaking/formation of O—H bond provides major contribution to charge storage while cation insertion/extraction playing a much less important role, thus offering important insight into energy storage behavior of NiO_2_H*_x_*. The systematic correlation between structural changes and the electrochemical charge storage properties can be used to probe any changes in charge storage mechanisms if the structure of NiO_2_H*_x_* is modified (e.g., doping of heteroatoms) to enhance electrochemical performance and versatility. Moreover, the general methodology of in operando*/*in situ spectroscopic technique demonstrated in this study is applicable to probing the fundamental relationship between material structure and functionality for other chemical and energy transformation processes.

## Supporting information

As a service to our authors and readers, this journal provides supporting information supplied by the authors. Such materials are peer reviewed and may be re‐organized for online delivery, but are not copy‐edited or typeset. Technical support issues arising from supporting information (other than missing files) should be addressed to the authors.

Supplementary
