# Supplementary material for: Probing Structural Evolution and Charge Storage Mechanism of NiO2Hx Electrode Materials using In Operando Resonance Raman Spectroscopy
Source: Adv Sci (Weinh). 2016 Feb 23;3(6):1500433. doi: 10.1002/advs.201500433 (PMC5067601; doi:10.1002/advs.201500433)
Supplement: Supplementary file 1 — Supplementary [file ADVS-3-0j-s001.pdf]

Copyright WILEY-VCH Verlag GmbH & Co. KGaA, 69469 Weinheim, Germany, 2016.

ADVANCED SCIENCE
Open Access

Supporting Information

for *Adv. Sci.*, DOI: 10.1002/advs.201500433

Probing Structural Evolution and Charge Storage Mechanism of NiO_2H_x Electrode Materials using In Operando Resonance Raman Spectroscopy

*Dongchang Chen, Xunhui Xiong, Bote Zhao, Mahmoud A. Mahmoud, Mostafa A. El-Sayed, and Meilin Liu**

Copyright WILEY-VCH Verlag GmbH & Co. KGaA, 69469 Weinheim, Germany, 2013.

Supporting Information

Probing structural evolution and charge storage mechanism of NiO_2H_x electrode materials using *in operando* resonance Raman spectroscopy

*Dongchang Chen, Xunhui Xiong, Bote Zhao, Mahmoud A. Mahmoud, Mostafa A. El-Sayed and Meilin Liu**

1. Experimental section:

1.1 Fabrication of NiO_2H_x thin film model electrode

All chemicals were all of analytical grade and were used without further purifications. To prepare NiO_2H_x thin film model electrode, the bare Ni foil (Alfa Aesar, 0.1 mm thickness) was electrochemically oxidized in a standard three-electrode cell in a 2 M KOH electrolyte. The Ni foil substrate, a standard Ag/AgCl electrode, and a Pt mesh were used as working electrode (WE), reference electrode (RE), and counter electrode (CE), respectively. The potential of WE was cycled between 0-0.6 V (vs Ag/AgCl) for 3000 cycles with a scan rate for 50 mV s^{-1}. After this process, the produced film was rinsed using DI water and was dried at 80 ^{o}C for 2 h. To evaluate the mass loading, thin coating was removed by sonicating the film under 0.1 M H_2SO_4. On the basis of accurate mass measurement, the mass loading of the NiO_2H_x is approximately 0.0117 mg cm^{-2}.

1.2 Electron microscopy characterizations

The morphology of NiO_2H_x model electrode was analysed using a scanning electron microscope LEO 1530. The transmission electron microscopic (TEM) image and SAED pattern was collected using a high resolution FEI Tecnai F30 transmission electron microscope.

1.3 Raman spectroscopic measurement

Raman spectra were obtained using a Renishaw RM 1000 spectromicroscopy system with a 20x/0.40 objective. An air-cooled Ar ion Laser (Mellos Griot) with wavelengths of 514 nm and 488 nm and a He-Ne Laser (Thorlab HRP-170) with wavelength of 633 nm were used as excitation of Raman spectra. The confocal slit was adjusted to be 5 um to minimize the band broadening effect due to the contribution of non-confocal signal.

1.4 Reflection spectroscopic measurement

The reflection spectroscopic measurement was performed using a reflection integration sphere (Ocean Optics ISP-50). An Ocean Optics DH-2000 light source, QP450-1 optical fiber, and a DH-2000 spectrometer were applied as light source, light transmission fiber, and detector respectively. The reference spectrum was acquired using an Ocean Optics reference standard. The acquisition time was 65 s with boxcar value of 3 to smooth the spectrum. The absorption spectrum of the NiO_2H_x model electrode was obtained by comparing the reflection spectra of both reference standard and model electrode.

Also, the reflection spectra measurement was conducted through customization of Raman spectrometer for better signal-to-noise ratio. A halogen lamp was used for microscope white light illumination. The reflected light was collected by a high NA objective (50X/0.75) and was then guided to grating chamber. The absorption spectrum of the NiO_2H_x model electrode was also calculated by comparing the reflection spectra of both Ocean Optics reflection standard and the model electrode.

1.5 *In operando* Raman cell setup

An *in-operando* Raman cell was used to collect Raman spectra and perform electrochemical measurement at the same time. The detailed design of the *in operando* cell is shown in Figure S1

and was described in our previous work.[S1] The cell body was made of Teflon PTFE with two series of concentric holes with different diameters. A stainless steel rod wrapped by PTFE insulation tape (to prevent leakage) which was inserted to the bottom of the cell served as the electric connection of WE. A Pt mesh connected with a Pt wire was applied as counter electrode. The Whatman filteres (GF/D) were used as separators to separate WE and CE. Similar with CE, a reference electrode (FlexRef WPI instruments) which was wrapped by PTFE tube was inserted into the other side of the cell. The tip of the RE was located exactly near the edge of WE and CE. Both of the RE and CE were wrapped by PTFE tape to prevent leakage. On the basis of three-electrode configuration, a quartz window was adhered on a PTFE washer on the top of the cell. The cell cap was bolted on the cell body with PTFE O-rings to prevent evaporation of electrolyte.

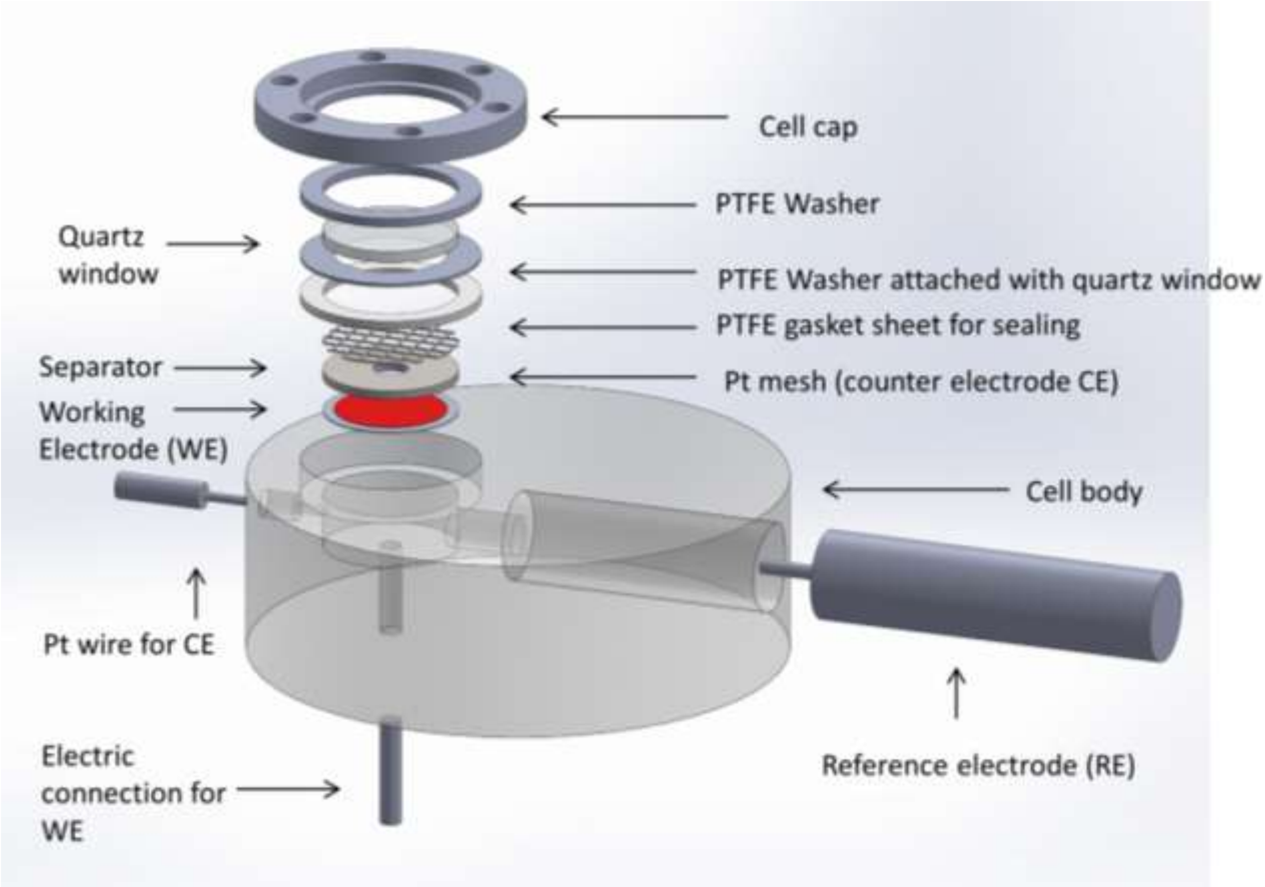

Figure S1. Construction of the three-electrode *in operando* Raman cell.

1.6 Electrochemical measurements

During *in operando* Raman spectroscopic acquisition, cyclic voltammetry (CV) measurements were performed at room temperature using an electrochemical workstation (Solartron, SI 1285) with a scan rate of 10 mV s^{-1} for 12 cycles. Raman spectroscopic acquisition time for each spectrum is 5 s (including the time for CCD camera response) and each Raman spectrum is acquired within 0.05 V consequently. The Raman spectrum of each potential in different cycles (totally 12 cycles) were then accumulated to improve the signal to noise ratio.

The charge-discharge experiments with different current densities were performed also using Solartron SI 1285. CV measurements under different scan rates was conducted using an electrochemical workstation (PARSTAT MC 1000).

2. Formation of NiO_2H_x thin film model electrode

When the bare Ni foil was immersed in 2 M KOH electrolyte, a thin layer of nickel hydroxide will be formed on the surface, because Ni metal starts to be unstable as long as the potential of Ni is higher than -0.7 V vs Ag/AgCl in strong base solution according to the Pourbix diagram of $Ni/NiO_2H_x/NiO_2$.[S2, 3] During extensive cycling of the Ni foil between 0 V and 0.6 V, the foil surface will be gradually oxidized to form γ-NiOOH eventually.[S3-5] Figure S2 shows the CV profile of the electrochemical oxidation of Ni foil within 3000 cycles. The foil exhibited negligible current density in the first cycle. However, it is obvious that the current density gradually increases with cycles numbers and doesn’t change significantly when the cycle number approach 3000. It is also noted that the over potential of the redox peak gradually increase with current density, which is due to the increased thickness of the nickel hydroxide film caused higher ohmic resistance.

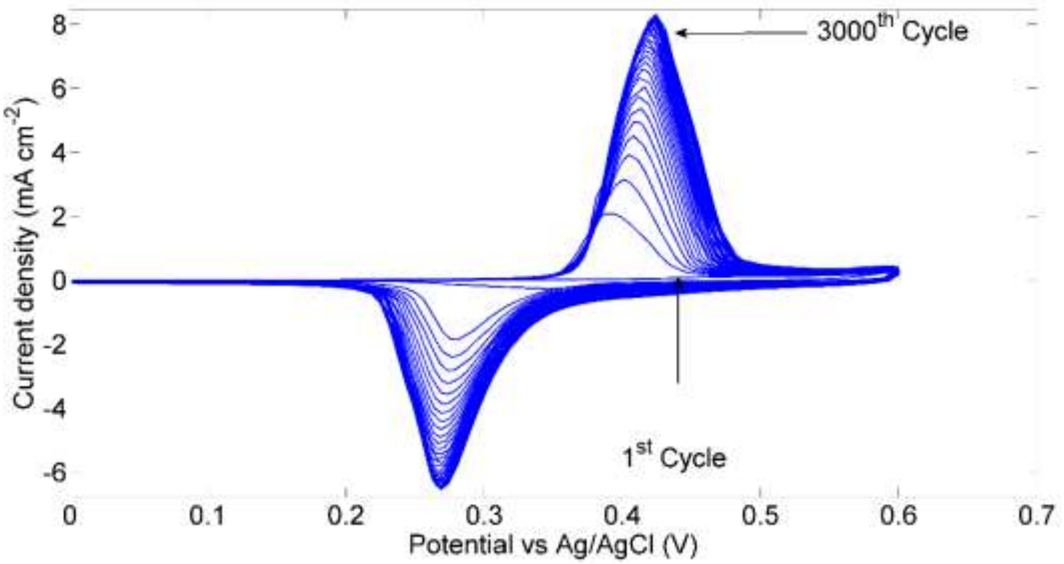

Figure S2. CV profiles of Ni foil within 3000 cycles with an interval of 50 cycles. The scan rate was 50 mV s^{-1}.

3. SAED pattern of NiO_2H_x thin film model electrode

Due to the limited thickness of the NiO_2H_x film, the X-ray diffraction pattern of the film barely exhibited diffraction signal of NiO_2H_x. Therefore, we applied TEM for the phase analyses. Figure 1c shows the TEM image of the scraped thin film of NiO_2H_x, which exhibits clear layered fringes. The SAED pattern is shown in Figure S3 which indicates three weak diffraction rings. Based on the XRD patterns of layer-structured γ-NiOOH based complex (JCPDS 23-1407, JCPDS 27-0764, JCPDS 06-0075) and the sizes of the diffraction rings shown in Figure S3, [S6, 7] we assign the three diffraction rings in the SAED pattern as the 001, 002, and -201 diffractions of γ-NiOOH ($C_{2/m}$ space group).

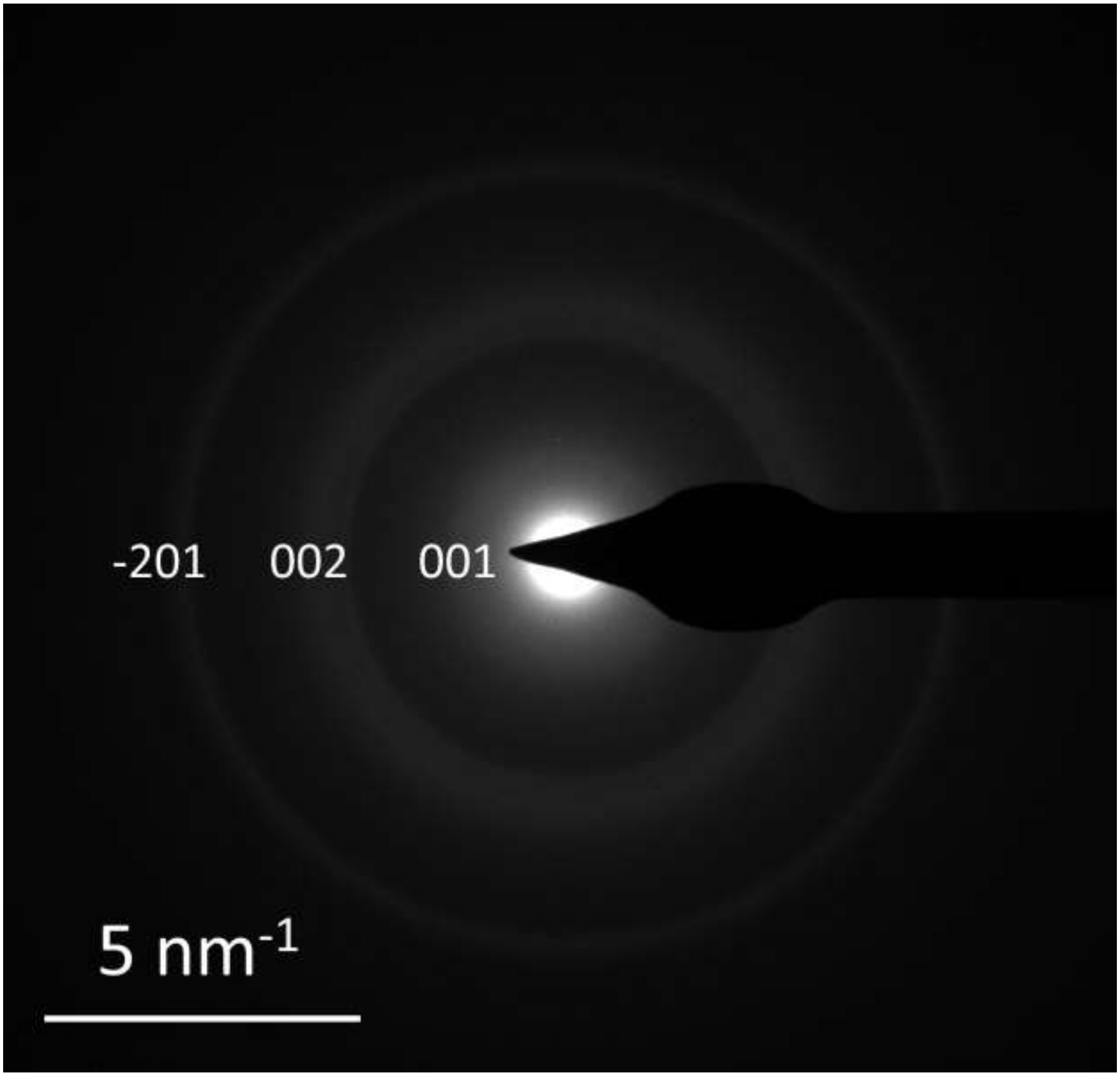

Figure S3. SAED pattern and diffraction ring assignments of scraped NiO_2H_x thin film.

4. Raman bands assignment

As mentioned in the main text, the layer-structured NiO_2H_x has complicated structure and stoichiometry, regarding to the amount of the bonded hydrogen and interlayer ions. Depends on the bond length and coordination of oxygen, the space group of the NiO_2H_x complex was reported as a variety of types (e.g. hexagonal R_{-3m} or monoclinic $C_{2/m}$).[S4, 6-8] In order to simplify

the phonon band assignment, the simple model of NiO_2 layer with highest order of symmetry ($R_{\bar{3}/m}$) was applied, which doesn't take the bonded hydrogen and interlayer ions into account (Figure S4).

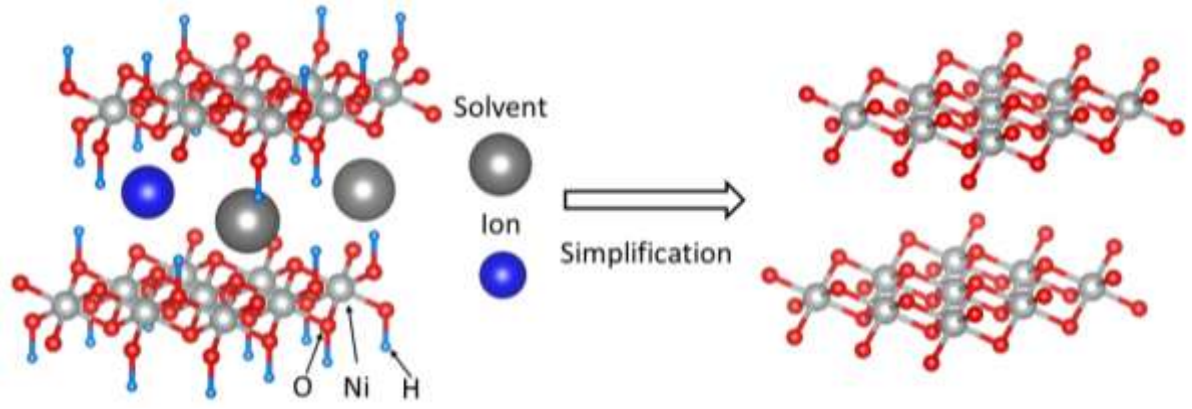

Figure S4. Illustration of simplification of NiO_2 layer structure model for Raman band assignment based on group theory.

Based on group theory analyses, the irreproducible representation of the NiO_2 layer can be written as:

$$\Gamma = A_{1g} + 2A_{2u} + 2E_u + E_g \qquad (1)$$

One A_{1u} and one E_u mode are acoustic modes. Among the four optical modes, the A_{1g} and E_g mode are Raman active, which basically match the experimental results that only two Raman bands were observed. For the A_{1g} mode, the oxygens vibrate perpendicular to the plane formed by oxgyen; whereas the oxygens vibrate along this plane for the E_g mode (Figure 1f). The Raman tensors of the two modes are:

$$A_{1g} = \begin{matrix} a & 0 & 0 \\ 0 & a & 0 \\ 0 & 0 & b \end{matrix} \quad E_{g1} = \begin{matrix} c & 0 & 0 \\ 0 & -c & d \\ 0 & d & 0 \end{matrix} \quad E_{g1} = \begin{matrix} 0 & -c & -d \\ -c & 0 & 0 \\ -d & 0 & 0 \end{matrix} \qquad (2)$$

It is noted that the Raman tensor of A_{1g} mode is diagonal matrix, whereas the Raman tensor of E_g mode has non-diagonal elements. Therefore, the scattered light of the A_{1g} mode will largely

maintain the polarization direction of the incident laser and the scattered light of the E_g mode will be depolarized due to the polycrystalline nature of the film. By switching the polarization configuration from $\bar{Z}(XX)Z$ to $\bar{Z}(XY)Z$, the relative intensity of the A_{1g} mode is greatly reduced, confirming the Raman band assignments and the validity to apply the simple NiO_2 layered model to approximate the structure of NiO_2H_x.

5. Absorption spectrum of NiO_2H_x model electrode

The absorption spectrum of NiO_2H_x model electrode was obtained by comparing the reflection spectra of a reference standard and the model electrode. Since there is no light transmission for the NiO_2H_x model electrode, the absorbance can be expressed by the following equation (R is reflectance):

$$Absorbance = 1 - {R_{sample}}/{R_{refenece}} \qquad (3)$$

Figure S5 shows the reflection spectra of both reference standard and the model electrode measured by the customization of Raman spectrometer using white light illumination (experimental section). The reflection spectrum of the reference standard describes the intensity profile of the light source used for white light illumination. The reflection spectrum of the model electrode indicates strongly reduced reflectance in the visible light range, especially in the region between 600-700 nm where a clear reflectance minimum is observed. The resulting absorption spectrum shows wide absorbance profile in the visible light range with a maximum between 650-700 nm (Figure S6a and Figure 1e).

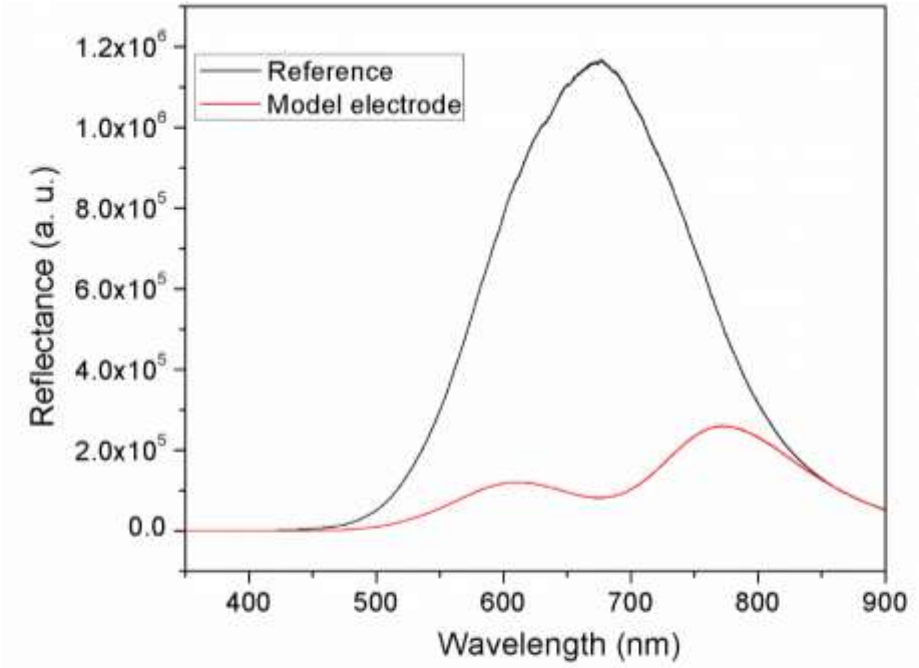

Figure S5. Reflection spectra of an Ocean Optics reference standard and the NiO_2H_x model electrode acquired by customization of Raman spectrometer using white light illumination.

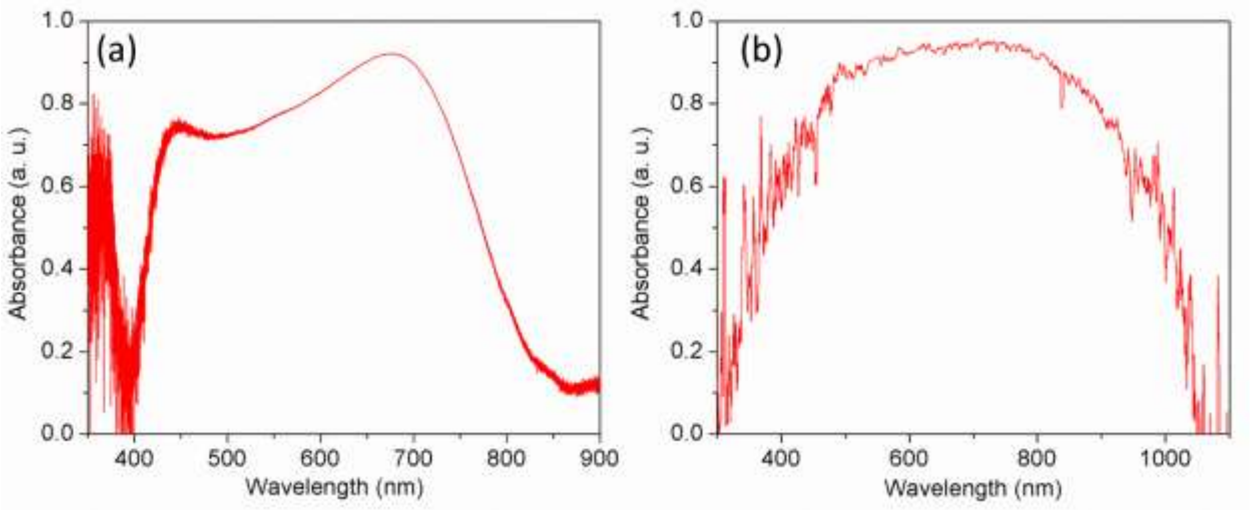

Figure S6. The absorption spectra acquired by (a) the customization of Raman spectrometer using white light illumination and (b) Ocean Optics UV-vis spectrometer equipped with an integration sphere.

Since the reflection spectra acquired by the customization of Raman spectrometer was collected by an objective (50x/0.75), a small portion of reflected light cannot be collected. Therefore, a standard Ocean Optics UV-vis spectrometer equipped with an integration sphere was applied to

the measurement of the reflection spectra. Due to the limited efficiency of integration sphere and much lower sensitivity of CCD detector of UV-vis spectrometer than that of Raman spectrometer, the signal-to-noise ratio of the absorption spectra (Figure S6b) acquired by Ocean Optics is much lower than that of the spectra acquired by customization of Raman spectrometer (Figure S6a). However, it is obvious that the two spectra show same absorption characteristics generally, which is the broad absorbance profile with a maximum near 700 nm.

6. Raman spectra band fitting

The Raman fitting calculation of each Raman spectra were done by assuming a linear baseline and that all Raman bands have a Lorentzian line shape:

$$F(\nu) = \frac{I*\Gamma}{(\nu-\nu_0)^2+\Gamma^2} \tag{4}$$

In this equation, ν is the Raman shift; I is Raman band intensity, ν_0 and Γ are position and FWHM of Raman band, respectively. The band fitting process was conducted through a Matlab fitting code automatically. Since only two bands were fitted (A_{1g} and E_g) and the positions of the two bands are well-separated, the automatic band fitting can ensure the accuracy for band evolution analysis.

7. The measurement of the reflectance under *in operando* conditions

In order to corroborate the analyses obtained from *in operando* Raman spectroscopic measurment, we performed the measurement of reflectance of the model electrode under *in operando* conditions. Light reflection is complementary to light absorption, which corresponds to the electronic structure of the materials (i.e. reflecting the oxidation state). Figure S7 shows the reflectance profile of the electrode as the potential (vs Ag/AgCl) of the working electrode (WE) was changed from 0 V to 0.5 V in a 2 M KOH electrolyte solution. A signficant red shift of the reflection profile (correpsonding to a blue shift of absorption) is clearly observed as WE

potential was switched from 0 V to 0.5 V. This shift suggests a increase in the energy gap of electronic state transition and evolution of the oxidation state of Ni, consistent with the Raman spectroscopic evolution as a function of electrochemical potential. It is noted that the reflection profile under the *in operando* condition shown in Figure S7 exhibited discrepancies with the light reflectance profile under ex-situ condition shown in Figure S5, since the existence of electrolyte will strongly interfere with light absorption/reflection. Also, the light reflectance profile under *in operando* condition cannot be converted to the light absorption profile due to lack of the light reflectance behavior of a reference standard under the same operando condition. However, the evolution of light reflectance alone can provide strong evidence of change in oxidation state during cell operation.

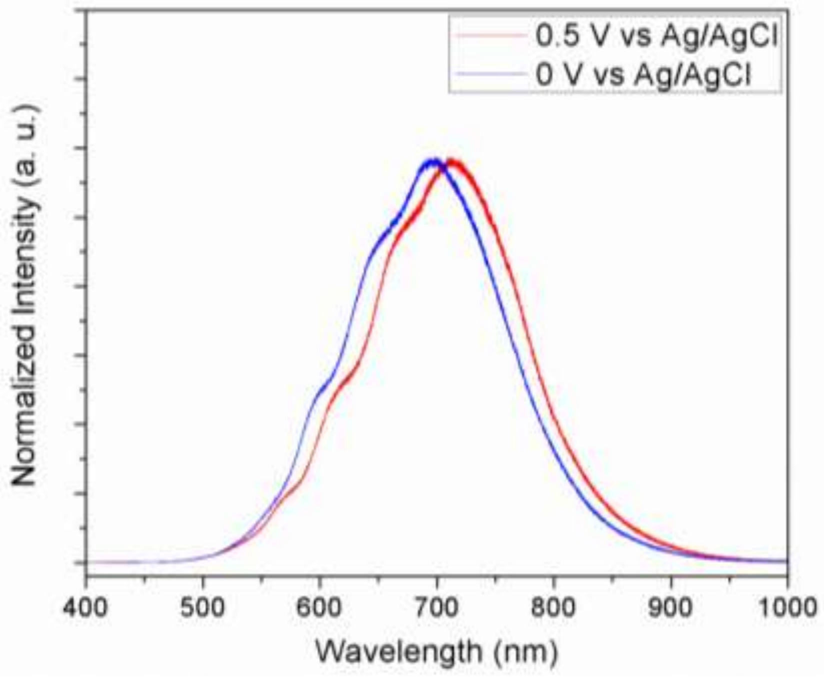

Figure S7. Light reflection measurement of NiO_2H_x thin film model electrode at 0 V and 0.5 V in a 2 M KOH aqueous electrolyte solution.

8. Electrochemical behavior of NiO_2H_x model electrode

Figure S8 shows the CV profiles of NiO_2H_x model electrode tested in 2 M KOH electrolyte with different scan rates (the potential window for 500 mV s^{-1} was expanded). It is obvious that the CV profile was well-maintained under high rate operation. Similarly, the charge-discharge experiment with different current densities was also performed to calculate the capacitance/capacity retention (Figure S9 and S10). At a high current density of 5 mA cm^{-2} (427 A g^{-1} with respect to the mass loading of the active material; a charge/discharge cycle is finished within 5 seconds), the capacitance/capacity can be maintained more than 80 %. These experimental facts indicate the flat geometry of NiO_2H_x model electrode can greatly reduce the kinetic hindrance caused by electrolyte mass transport, which is crucial for unambiguous and quantitative correlation between phonon properties and electrochemical features.

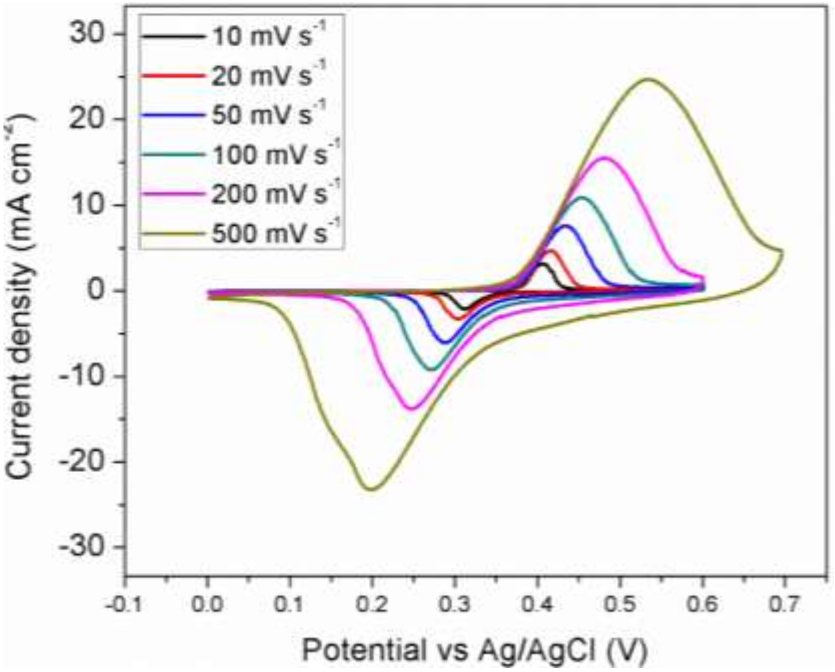

Figure S8. Cyclic voltammogram (CV) of NiO_2H_x thin film model electrode with different scan rates in a 2 M KOH aqueous electrolyte.

As suggested by Simon et al. and Brousse et al.,[S9, 10] the electrochemical chemical behavior with distinctive and abrupt CV peaks belongs to a typical battery behavior. Therefore, the units

for the charge storage of battery system (Ah or C) were used for charge storage capacity calculation. The calculated areal and specific capacity are 10.98 mC cm^{-2} and 261 mAh g^{-1} (or 1,877 F g^{-1}), respectively (considering the fact that a large number of reported works about battery-like materials have already used Farad as unit, the charge storage capacity calculated using the unit "F g^{-1}" is 1877 F g^{-1}).

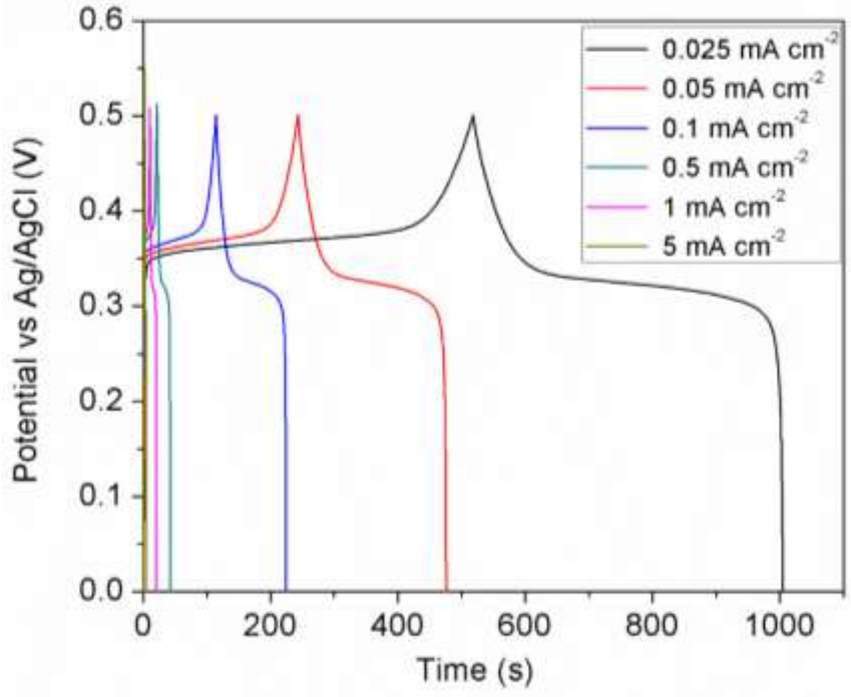

Figure S9. The charge-discharge profiles of NiO_2H_x model electrode with different current densities in a 2 M KOH aqueous electrolyte.

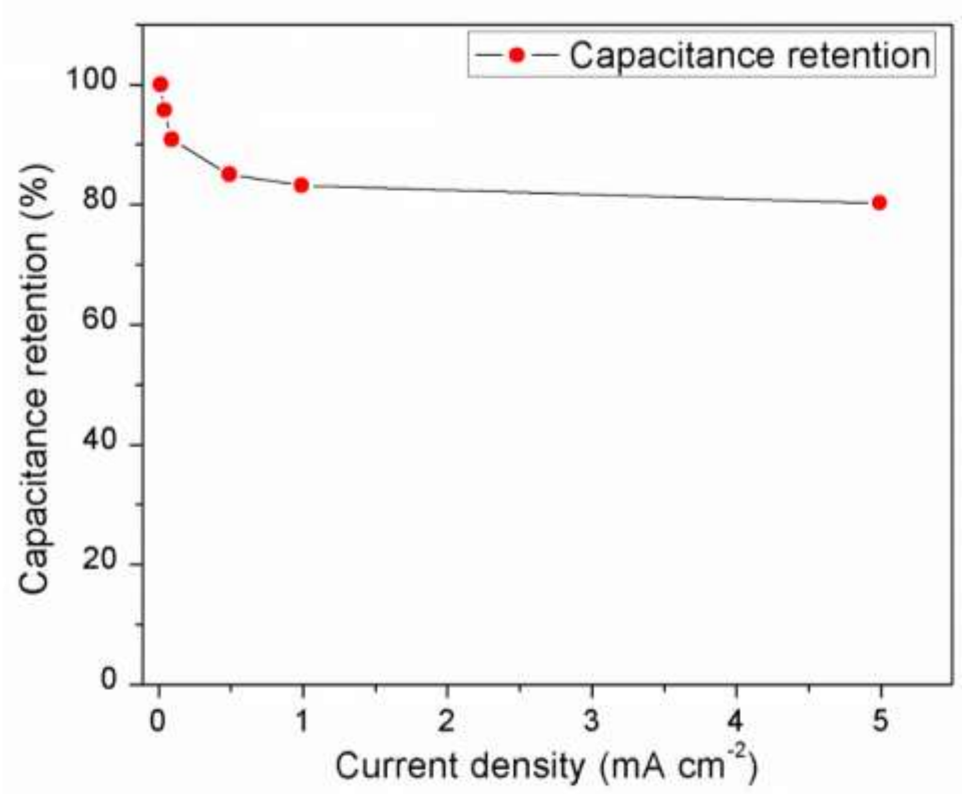

Figure S10. The capacity/capacitance retentions of NiO_2H_x model electrode tested in 2 M KOH aqueous electrolyte as functions of current densities.

The CV profile NiO_2H_x model electrode tested in 2 M KNO_3 shows a rectangular-like shape and can be well maintained under high rate operation (500 mV s^{-1}, Figure S11), which is similar as most of cation incorporation-based pseudocapacitive charge storage (e.g. MnO_2).[S1]

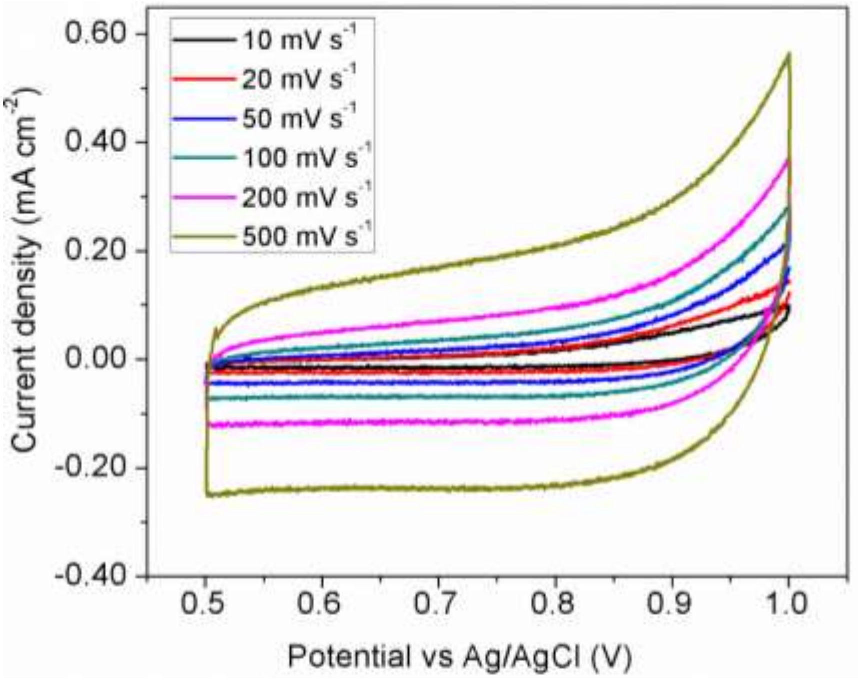

Figure S11. Cyclic voltammogram (CV) of NiO_2H_x thin film model electrode with different scan rates in a 2 M KNO_3 aqueous electrolyte.

9. Calculation of charge density as a function of electrochemical potential

The charge densities as a function of electrode potential shown in Figure 4 were integrated from the CV curves shown in Figures 2 and 3. The relationship between the CV current and the stored charge at a particular time (t_0) is expressed by the following equation.

$$Q(t_0) = \int_0^{t_0} i(t)dt \tag{5}$$

where Q is the stored charge density in C cm^{-2}, $i(t)$ is the CV current density in A cm^{-2}.

The CV profile shown in Figure 2 and the integrated charge density (shown in Figure 4d) are compared in Figure S12. At the highest electrochemical potential, the stored charge is maximized.

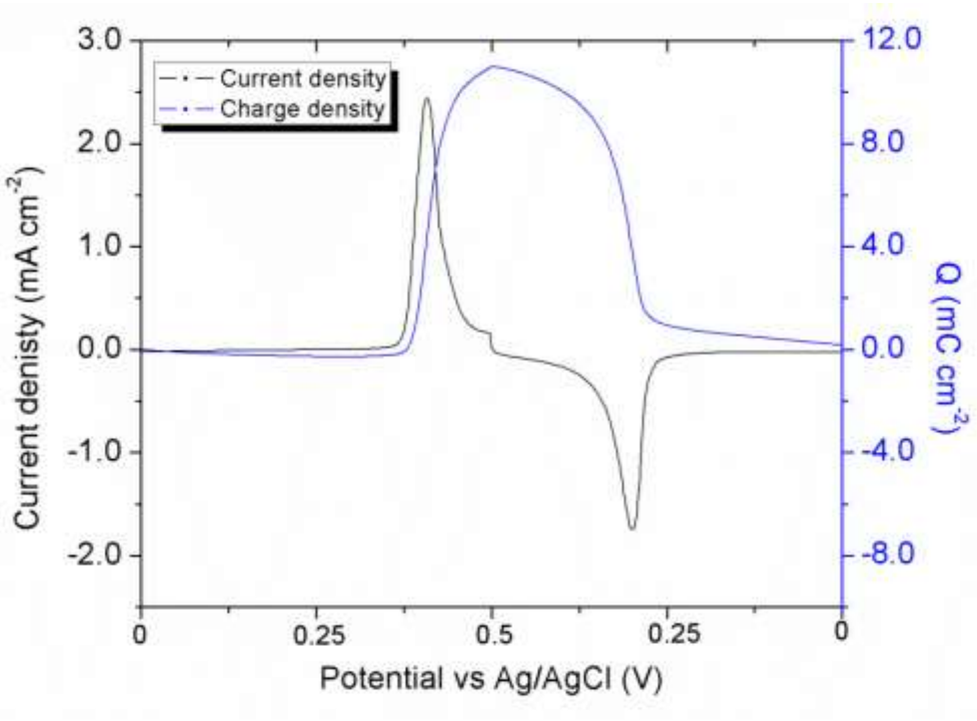

Figure S12. The comparison of the CV profile shown in Figure 2 and the charge density shown in Figure 4d.

10. *In operando* Raman spectroscopic evolution under galvanostatic charge/discharge

In order to compare the spectroscopic evolution of NiO_2H_x under the conditions for cyclic voltammery shown in Figure 2, similar *in operando* experiments were conducted under galvanostatic charge/discharge. In cyclic voltammetry, the rate of electrochemical reations increases with the potential and reaches the maximum at the electrochemical potential where the redox peaks are observed. In contrast, the rate of electrochemical reactions is relatively constant under galvanostatic charge/discharge; the structural changes induced by electrochemical reacitons are more gradual. Figure S13 shows the *in operando* Raman spectroscopic evolution acquired at a constant current density of 50 µA cm^{-2}. As we expected, the evolution of Raman spectra was more gradual under galvanostatic charge/discharge conditions, not abrupt as seen under CV conditions, implying that the electrochemical current leads to the structural changes.

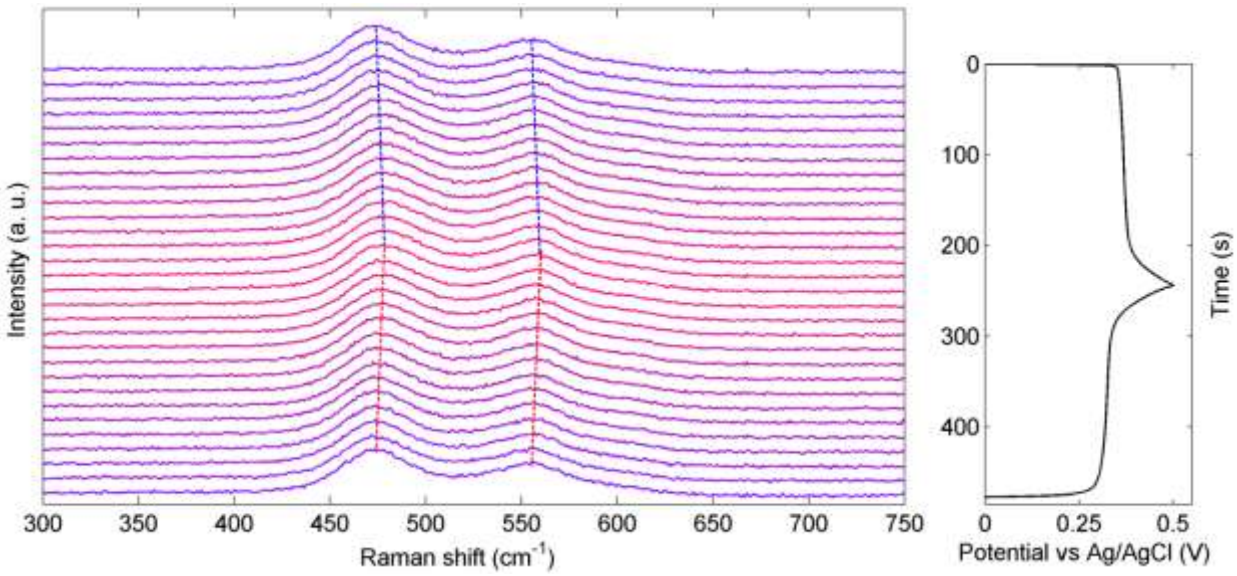

Figure S13. The Raman spectroscopic evolution of NiO_2H_x model electrode acquired at a constant current density of 50 µA cm^{-2} in a 2 M KOH electrolyte between 0 V and 0.5 V.

11. Effects of electrolyte cations

In order to evaluate the effects of the types of the cations on the electrochemical behavior and the structural changes during cycling of the NiO_2H_x model electrode, we performed the same electrochemical and *in operando* Raman measurements on the NiO_2H_x electrodes from the same batch as the electrolyte solution was changed from KOH to NaOH and LiOH. Since the interlayer spacing of the NiO_2H_x family material ranges from 5 to 7 Å,[S11] intercalation of different types of cations such as Li^+ (0.59 Å), Na^+ (0.99 Å), and K^+ (1.37 Å) may influence the electrochemical behavior and charge storage mechanisms of the electrode. Figure S14 shows the CV profiles of the NiO_2H_x model electrode tested in 2 M aqueous electrolyte solution of LiOH, NaOH, and KOH. It is found that the evolution of *in operando* Raman spectra remained relatively the same when electrolyte of different type of cations was used, indicating that the charge storage mechanism (breaking/formation of O-H bond) is independent of the types of cations. However, the electrochemical behavior (e.g., CVs) of the NiO_2H_x model electrodes changed slightly as the type of electrolyte cations was changed from one type to another. For

example, the redox peak positions in the CVs were shifted slightly to higher potentials as the electrolyte cations were changed from K^+ to Na^+ and to Li^+, due most likely to stronger solvation effect for smaller cations.

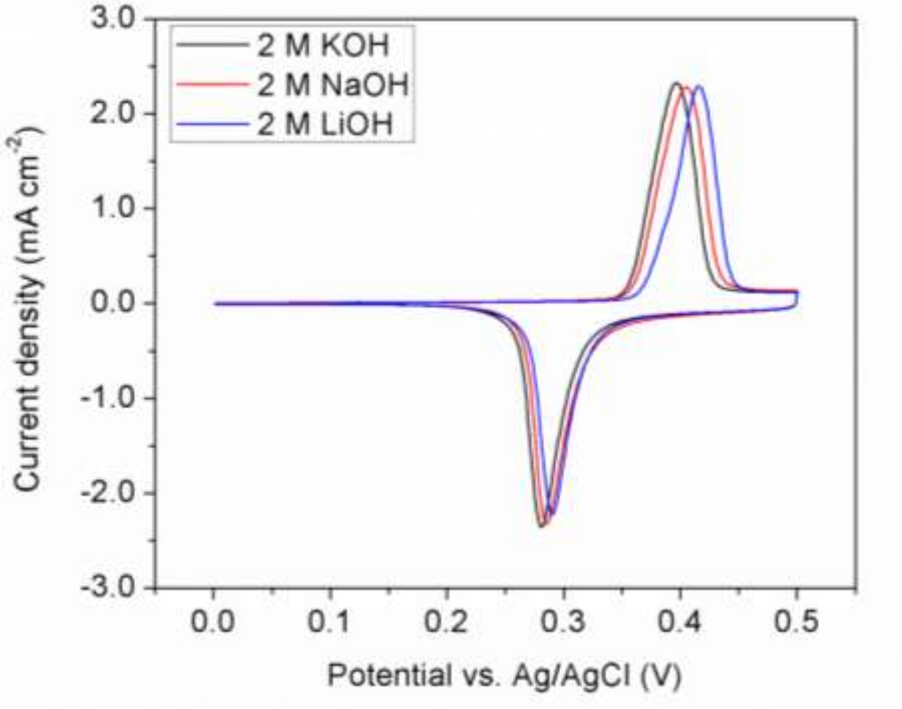

Figure S14, Cyclic voltammograms (CV) of NiO_2H_x thin-film model electrodes tested in 2 M aqueous electrolyte solution of LiOH, NaOH, and KOH. The scan rate is kept at 10 mV s^{-1}.

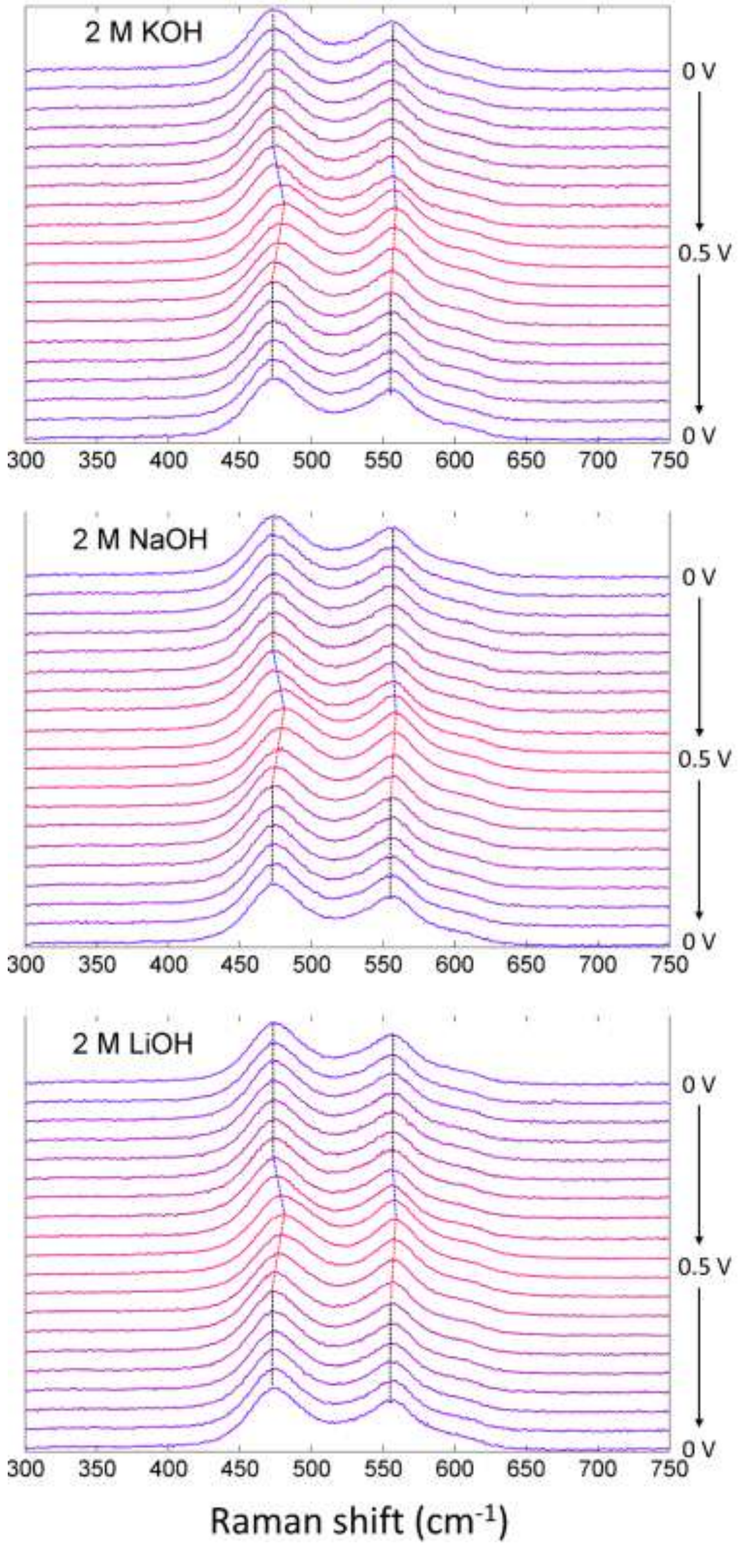
2 M KOH
0 V
0.5 V
0 V
300
350
400
450
500
550
600
650
700
750
2 M NaOH
0 V
0.5 V
0 V
300
350
400
450
500
550
600
650
700
750
2 M LiOH
0 V
0.5 V
0 V
300
350
400
450
500
550
600
650
700
750
Raman shift (cm-1)

Figure S15. Evolution of *in operando* Raman spectra of a thin-film model NiO_2H_x electrode cycled in 2 M aqueous KOH, NaOH, and LiOH aqueous electrolytes. The experimental setting is exactly the same as described in Figure 2.

12. Raman spectra of NiO_2H_x in 2 M KNO_3 electrolyte

Figure S16 shows the comparison of the Raman spectra of as prepared NiO_2H_x model electrode and the NiO_2H_x model electrode immersed in 2 M KNO_3 electrolyte. It is obvious that the band profile changed considerably, especially the band width of ν_2 band (E_g). The band broadening is a clear indication of cation incorporation between the interlayer spacing, because the incorporated cations will lead to break of the lattice symmetry, leading to structural disorder and thus band broadening effect.[S1, 12-14] Similar band broadening effects related to cation incorporation has been reported in different works related to lithium ion battery and pseudocapacitive charge storage.[S1, 12-14] Therefore, Raman spectroscopic analyses clearly indicate that NiO_2H_x can incorporate cations, but the cation incorporation can’t form massive charge storage proven by electrochemical and Raman spectroscopic analyses.

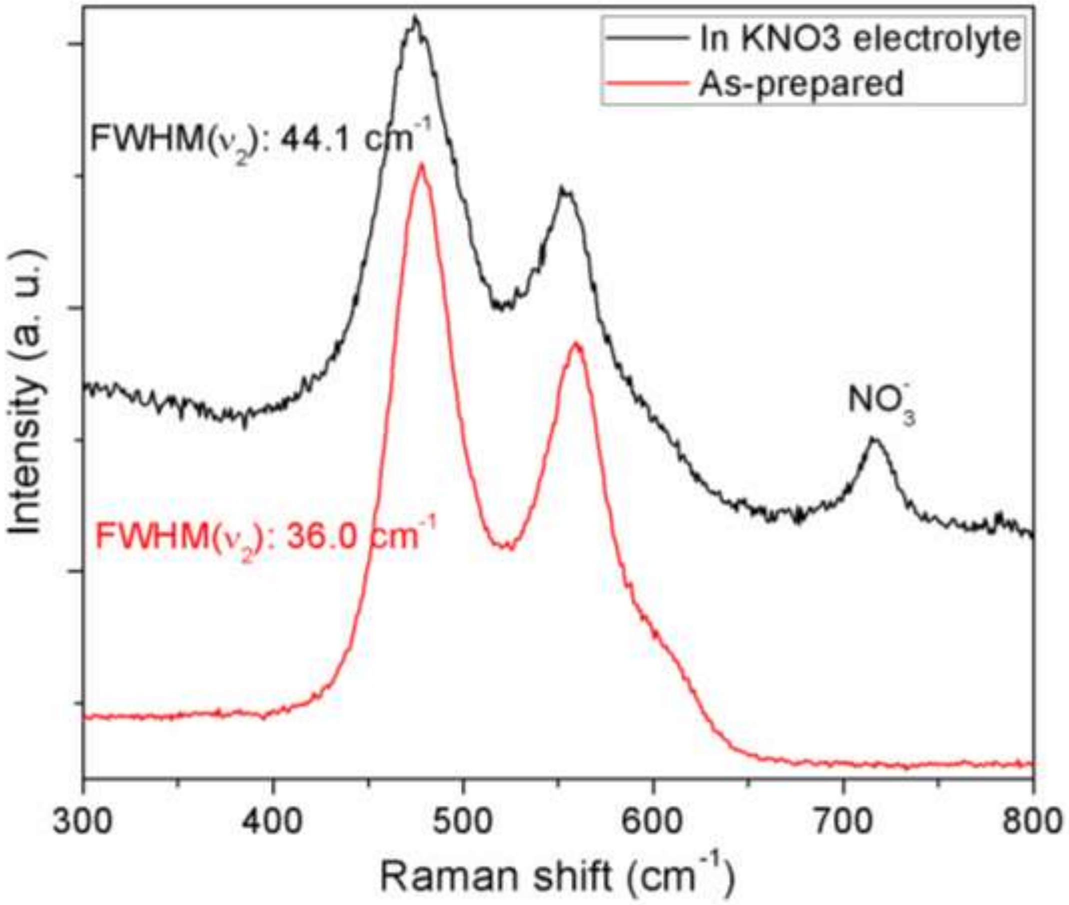

Figure S16. Comparison of the Raman spectra of as-prepared NiO_2H_x model electrode and the electrode immersed in 2 M KNO_3 electrolyte at OCV condition.

Reference

[S1] D. Chen, D. Ding, X. Li, G. H. Waller, X. Xiong, M. A. El-Sayed, M. Liu, *Chem. Mater.* **2015**, *27*, 6608-6619.

[S2] B. Beverskog, I. Puigdomenech, *Corros. Sci.* **1997**, *39*, 969-980.

[S3] C. A. Melendres, S. Xu, *J. Electrochem. Soc.* **1984**, *131*, 2239-2243.

[S4] H. Bode, K. Dehmelt, J. Witte, *Electrochim. Acta* **1966**, *11*, 1079-IN1071.

[S5] D. J. Jeong, W.-S. Kim, Y.-K. Choi, Y.-E. Sung, *J. Electroanal. Chem.* **2001**, *511*, 79-87.

[S6] H. Bode, K. Dehmelt, J. Witte, *Z. Anorg. Allg. Chem.* **1969**, *366*, 1-21.

[S7] O. Glemser, J. Einerhand, *Zeitschrift für anorganische Chemie* **1950**, *261*, 43-51.

[S8] H. Bartl, H. Bode, G. Sterr, J. Witte, *Electrochim. Acta* **1971**, *16*, 615-621.

[S9] P. Simon, Y. Gogotsi, B. Dunn, *Science* **2014**, *343*, 1210-1211.

[S10] T. Brousse, D. Bélanger, J. W. Long, *J. Electrochem. Soc.* **2015**, *162*, A5185-A5189.

[S11] P. Oliva, J. Leonardi, J. F. Laurent, C. Delmas, J. J. Braconnier, M. Figlarz, F. Fievet, A. d. Guibert, *J. Power Sources* **1982**, *8*, 229-255.

[S12] K. Dokko, Q. Shi, I. C. Stefan, D. A. Scherson, *J. Phys. Chem. B* **2003**, *107*, 12549-12554.

[S13] M. Inaba, Y. Iriyama, Z. Ogumi, Y. Todzuka, A. Tasaka, *J. Raman Spectrosc.* **1997**, *28*, 613-617.

[S14] C. Julien, M. Massot, *Phys. Chem. Chem. Phys.* **2002**, *4*, 4226-4235.
